# The Cholera

**Published:** 1848-07

**Authors:** 


### THE MEDICAL EXAMINER.

PHILADELPHIA, JULY, 1848.

### THE CHOLERA.

It was hoped that the further progress of the Cholera on the Continent of Europe was stayed, but the last steamer from England brings intelligence of a reappearance of the disease in Moscow and other parts of Russia, marked by great severity.

